# Effects of Humor in Health Communication: Experimental Evidence for Video Sequences Aiming to Increase the Willingness to Donate Organs

**DOI:** 10.3389/fpubh.2021.651736

**Published:** 2021-06-24

**Authors:** Rebecca Baumeister, Florian Fischer

**Affiliations:** ^1^School of Public Health, Bielefeld University, Bielefeld, Germany; ^2^Institute of Public Health, Charité – Universitätsmedizin Berlin, Berlin, Germany; ^3^Institute of Gerontological Health Services and Nursing Research, Ravensburg-Weingarten University of Applied Sciences, Weingarten, Germany

**Keywords:** humor, medical cabaret, organ donation, organ transplantation, testimonial, entertainment education

## Abstract

Humor has been proven to provide support when dealing with fear-related aspects of life. Therefore, it might be a useful communication strategy for addressing the need for donated organs. We conducted an experimental study among University students in Germany (*n* = 236) aged about 25 years (M = 24.60, SD = 5.86) investigating the effects of four video sequences related to organ donation on the willingness to donate organs. Based on random allocation, each study participant received one video sequence. The video sequences were presented by either a prominent or non-prominent speaker and included either humorous or neutrally framed information related to organ donation. An online survey was conducted before and after the intervention. A follow-up to investigate long-term effects was conducted 4–6 weeks later. Overall, the four interventions led to high proportions of self-reported willingness to think about organ donation and talk about it with relatives and friends subsequent to the respective intervention. Appraisals beneficial to organ donation improved significantly only in the non-humorous interventions. It seems to be of little relevance whether the humorous message was communicated by a prominent or non-prominent person. However, future investigations should focus on sample populations with lower education, because they are less likely to possess an organ donor card and more likely to have lower levels of positive attitudes toward organ donation and may, therefore, show different results regarding the effectiveness of humorous interventions.

## Introduction

Referring to the Oxford English Dictionary, humor is defined as “that quality of action, speech, or writing which excites amusement” (1). From a psychological perspective, humor is a rather broad, multifaceted, and complex phenomenon (2). In relation to health, evidence shows that humor can relieve stress and improve quality of life (3). It may transform fears and other negative emotions into positive emotions expressed through laughter (4). However, there are various forms of humor, spanning a range from adaptive to maladaptive humor (5). According to this perspective, four styles of humor can be broadly distinguished: Affiliative, self-enhancing, aggressive, and self-defeating (6). These styles have varied impacts on health, which are also mediated through personality characteristics (7).

Humor can involve a wide range of cognitive and behavioral functions (8, 9). Thus, it can have an impact on both social and emotional characteristics. For this reason, humor can help to deal with contradictory messages as well as fear-related subjects. It holds people's attention by engaging them in complex cognitive processes, such as dealing with incongruity. Humor is by nature confrontational—either cognitively, emotionally, or both (10). Hence, it might be a useful communication strategy for addressing the need for donated organs. Despite several ethical discussions about the organ donation system within society and politics in the recent past, an opt-in solution is still in place for postmortem organ donation in Germany. This means that people must actively provide their informed consent in the form of an organ donor card, if they want to be registered as potential organ donors. This organ donor card also offers the opportunity for card holders to reject organ donation or to transfer the decision to another person. However, there is a large mismatch between the numbers of organs needed and those available. Patients are even dying on waiting lists before a suitable donor organ can be provided for them (11, 12). Although communication campaigns have focused on these issues, they have mainly provided information in a neutral form. Therefore, a recommendation has been formulated for rethinking and switching from a “one-size-fits-all” campaign to community-based, targeted health messaging (13). One such strategy might include the use of humor, although until now only limited evidence has been available concerning its efficacy (14, 15). Furthermore, previous studies have focused on celebrity endorsement as a well-established marketing strategy, which can also be applied in health campaigns (16). Following this, it has been shown that an effective strategy is to engage people who are known to the public to provide testimonials to improve the impacts on appraisal, recall, and level of engagement related to health-related topics, such as in anti-smoking campaigns (17).

One might expect that humor could be used as a tool in health communication, particularly in communicating subjects which are either fear-related or go against our individual preferences, because it also engages our attention by surprising us with incongruity (18). This is particularly relevant in terms of promoting willingness to donate organs, because the attitude toward organ donation is affected by a variety of factors, such as sociodemographic characteristics, level of knowledge, and personal experiences, as well as cultural and societal factors (19). Furthermore, several factors determine the willingness to donate organs, and these can be distinguished into prodonation and antidonation (20). The main reasons for organ donation (*prodonation*) are related to perceived humanitarian benefits, altruism, and feelings of contentment and pride (19, 21). Fear is the main reason preventing people from donating organs (*antidonation*). Various fears relate to the concept of diagnosing irreversible brain function failure as the indication of death in humans, physical mutilation, inadequate medical care, mistrust in physicians and the institutions involved in transplantation medicine, as well as general anxiety or discomfort before one's death (19, 20).

Until now, research on humor in health communication has focused mainly on the implications of mass media (22). In this context, it was observed that humor in advertisements increased attention and privileged recognition. Humor has been shown to improve the persuasiveness of preventive messages. The relevance of mass media in the context of health communication stems from the fact that it reaches large numbers and heterogeneous groups of people (22, 23). A study by Sukalla et al. (24) found that the intention to share a health-related video online was increased when it included humorous appeals. Nevertheless, research on health communication has clearly shown that preventive health messages disseminated via mass media do not always have the desired effect. Many preventive health campaigns use fear appeals to persuade people to adopt healthy behaviors or reduce risky behaviors (25). However, triggering fear can lead to defensive reactions, such as avoidance or denial (26).

One way to circumvent the phenomenon that preventive messages are perceived as a threat to an individual's attitudinal freedom would be to introduce humor into health communication. It is expected that humor will reduce the intensity of the perceived threat that leads people to engage in counterarguments. Humorous messages generally have the potential to reduce health-based anxiety and, in turn, promote positive behavior (27). In contrast, McGraw et al. (28) have underlined that humorous messages can lead to decreased problem perception and problem-solving behavior, particularly when the message is perceived as non-serious. Furthermore, it has been shown that the effects of humor vary according to an individual's involvement in the problem, indicating that greater involvement is associated with a lower appreciation of the humorous message (29).

This study builds on the current state of knowledge in this area and focuses on the effects of humorous interventions when addressing aspects of organ donation. Some previous studies have already investigated the effects of humorous interventions in a live medical cabaret (14, 15). This study uses almost the same humorous intervention, but presented within a video sequence, and explores its effects. In a total of four video-sequence interventions, we investigate the effects of: (1) a humorous vs. neutral formulation and (2) the impact of a prominent vs. non-prominent speaker presenting the intervention.

## Methods

### Study Design and Participants

We compared four interventions in an experimental online study using a between-subjects design. A simple randomization was used to assign each study participant to one of these interventions directly at the beginning of the questionnaire, and, therefore, irrespective of any other item such as demographic characteristics or willingness to donate organs. All four interventions consisted of a video sequence on organ donation. Data were collected at three time points: before the intervention (t_0_), immediately after the intervention (t_1_), and ~4–6 weeks later (t_2_). An *a priori* sample size estimation indicated that 51 participants per group were needed to identify significant differences between two independent means, using a one-tailed test, assuming an effect size of *d* = 0.05, power β = 0.8 and a level of significance at α = 0.05.

The study population consisted of students who were enrolled at a University (including universities of applied sciences) in Germany at the time of the first survey (May 2019). A random selection was used to select 75 universities in Germany. Then, one faculty or department in each University was selected randomly, if more than one was available. A hyperlink to the online survey was emailed to the selected faculties and departments with a request to forward it to their students. We invited students to participate in an interventional study dealing with aspects of communication about health-related issues. The exact scope of the study was not explained to avoid any selection bias which may have occurred when explaining either the topic or the kind of intervention. At the end of the first survey (t_0_ and t_1_), participating students were asked to provide their email address, in order to be contacted for the second survey (t_2_). Email addresses were entered and stored separately from the study questionnaires in order to ensure the data privacy of study participants. To be able to merge the questionnaires from t_0_/t_1_ and t_2_, an individual code was created by each participant during the first survey, which then had to be entered on the second survey. All participants provided written informed consent before taking part in the study. The study received an ethical waiver from the ethics committee of Bielefeld University.

### Interventions

The four interventions each consisted of one video sequence on the topic of organ donation. The interventions differed in two characteristics: (1) Basically, we compared a humorous framing to a scientifically neutral message strategy. (2) A further distinction was that these two variations in presenting a statement on organ donation were conveyed by one prominent and one non-prominent person. In all four interventions, the same topics regarding organ donation were addressed (including numbers related to organ donation, organ donation cards, and fears and caveats regarding organ donation).

#### Humorous Interventions

The humorous intervention was based on a video sequence of a cabaret show on television, where the science journalist and famous German medical cabaret artist, Dr. Eckart von Hirschhausen, presented a sequence on organ donation (Intervention humor/prominent person: “I_Humor_Prom”). This sequence has also been part of Dr. Eckart von Hirschhausen's live medical cabaret “*Endlich!*” (a German pun, meaning both “at last” and “finite”). It sensitized the audience to fears related to organ donation and used the mechanism of incongruity, a characteristic of humorous messages, to debunk these fears. Furthermore, reasons for organ donation, such as solidarity and altruism, were addressed (14). This sequence has already been investigated in a previous study with regard to its effect on attitudes toward organ donation among a live audience of the medical cabaret [humorous intervention(s) vs. control] (14, 15), and also using an experimental laboratory design among students to test the humorous intervention vs. a neutral control (15). In our study, the same sequence as that provided by Dr. Eckart von Hirschhausen was also delivered by a non-prominent person; the pseudonym Dr. Tobias von Heldhausen was used for the speaker (Intervention humor/non-prominent person: “I_Humor_Non-Prom”). Comparable stylistic elements were used to ensure that the stimulus differed only in the person presenting the humorous intervention. The video sequence lasted ~4:30 min.

#### Neutral Interventions

The neutral video sequences contained the same kind of information as that presented in the interventions described above. However, the topic was presented in a neutral scientific manner, once by Dr. Eckart von Hirschhausen as the prominent person (Intervention neutral/prominent person: “I_Neutral_Prom”) and again by the non-prominent person with the pseudonym Dr. Tobias von Heldhausen (Intervention neutral/non-prominent person: “I_Neutral_Non-Prom”). The presentation took place without the use of any kind of humor and against the neutral background of a gray wall. Again, the recorded text was identical in both interventions. The duration of these video sequences was ~3:00 min each.

### Measures

A standardized and validated questionnaire already applied in the previous study by Heitland et al. (14) among the live audience was used. This questionnaire is based on theoretical considerations and empirical results related to the reasons for organ donation (prodonation) and fears and caveats regarding organ donation (antidonation). In total, the questionnaire contained 19 questions before the intervention (t_0_), 7 questions after the intervention (t_1_), and 23 questions after 4–6 weeks (t_2_) regarding the following areas:

(1) Sociodemographic characteristics
(2) Attitudes and willingness related to organ donation
(3) Predictors of organ donation (prodonation and antidonation)
(4) Appraisal of the interventions.

The first three areas were assessed at t_0_. The appraisal of the intervention—in addition to an item on general attitude toward organ donation (“rather positive,” “neutral,” “rather negative,” and “I have not yet considered the topic”)—was assessed at t_1_. The questionnaire at t_2_ consisted of the same components as already assessed at t_0_, except that the academic subject of study was not asked again. Furthermore, we assessed whether the study participants were able to remember the video sequence.

(1) Sociodemographic characteristics: at the beginning of the questionnaire, sociodemographic characteristics (age, gender, and academic subject of study) were assessed.
(2) Attitudes and willingness related to organ donation: This was followed by a question on general attitudes toward organ donation with a categorical four-level response option (“rather positive,” “neutral,” “rather negative,” and “I have not yet considered the topic”) and a question on possession of an organ donor card, whose dichotomous answer (“yes” vs. “no”) was used as a filter question for the related decision. For those who did possess an organ donor card, a choice could be made between “I agree in principle to organ donation (at least one organ),” “I do not agree to organ donation” and “I have delegated the decision to remove organs to another person.” Those who did not possess an organ donor card had the following choices: “I intend to get one soon,” “I would agree to an organ donation,” “I would not agree to an organ donation,” “I am not eligible to donate organs for medical reasons,” and “I have not yet made a decision.” According to this, the willingness to donate organs was classified as positive if (1) possession of an organ donor card and the decision “I agree to organ donation” were affirmed, or (2) if no organ donor card was available but individuals indicated that they agreed in principle to organ donation. Furthermore, the answering options “I have not yet considered the topic” and “I have not yet made a decision” were categorized as a neutral attitude toward organ donation.
(3) Predictors of organ donation: In the following section of the questionnaire, dimensions of prodonation and antidonation, as proposed by Parisi and Katz (20), were investigated. These items were addressed by either humorous or neutral statements within both interventions. Statements related to these issues were formulated either positively or negatively (see **Tables 2**, **3**). The study respondents could indicate their agreement or disagreement by means of predetermined grading. All 13 items (five for prodonation and eight for antidonation) were surveyed on a six-point Likert scale (“strongly disagree,” “disagree,” “tend to disagree,” “tend to agree,” “agree,” and “fully agree”).
(4) Appraisal of the interventions (t1): The appraisal of the interventions included items on the entertaining nature of the respective interventions on a Likert scale ranging from 1 (“not at all entertaining”) to 10 (“very entertaining”). To measure self-reported short-term effects, we differentiated (1) whether participants gained new information about organ donation from the video sequence, (2) whether they wanted to think about organ donation in the near future, and (3) whether they wanted to talk with relatives and/or friends about organ donation.

### Statistical Analyses

All analyses were carried out using the statistical software SPSS version 25. The data sets for the survey time points t_0_/t_1_ and t_2_ were merged using the individualized codes of study participants. A significance level of 5% was chosen for all tests. All variables in the online-based study were mandatory so that we had no issue of missing values.

Descriptive statistics were used to characterize the sample. The subsequent non-response analysis, based on a Chi-square test and Fisher's exact test, referred to the measurement time points t_0_/t_1_ and t_2_. It was examined whether the individuals who participated in the survey at both measurement time points differed from those who participated only at the first survey time point with respect to age, gender, general attitude toward organ donation, possession of an organ donor card, willingness to donate organs, or the intervention.

Following Parisi and Katz (20), one prodonation and one antidonation scale was formed from the respective items for each scale for further analysis in order to compare their means at different time points. To determine internal consistency, Cronbach's alpha was calculated based on the data before (t_0_) and following the intervention (t_2_) for the total sample. The internal consistency of the Cronbach's alpha indices ranged from values of 0.743 to 0.807. There was only slight improvement in internal consistency for both scales at both time points when individual variables were excluded. Therefore, all variables were included to form the respective sum indices. For significance testing of the prodonation and antidonation scales in relation to the willingness to donate organs, the Mann-Whitney *U*-test was applied as a non-parametric procedure.

The effects of the intervention on organ-donation-related attitudes were considered to be significant differences between the measurement time points. In the evaluation, both the changes in individual items and changes in the sum indices were considered. The Wilcoxon signed-rank test as a non-parametric statistical hypothesis test was used to test the significance of the ordinal scaled variables. This referred to the variables “General attitude toward organ donation” at the two respective time points [short-term effects (t_0_ to t_1_) and long-term effects (t_0_ to t_2_)] and differentiated by intervention groups. The McNemar test was applied as a non-parametric procedure for the observation of the dichotomous characteristics of the sample. For the appraisal of entertainment within the four intervention groups, we used an ANOVA test to describe significant differences. Changes in the mean scores on the prodonation and antidonation scales between survey time points were examined using a paired samples *t*-test. Cohen's d was determined as a measure of effect size, where a value below 0.5 indicates a small effect, a value between 0.5 and 0.8 a medium effect, and higher than 0.8 a large effect. To investigate the effects of the intervention, we focused on those participants who completed both t_0_/t_1_ and t_2_.

## Results

### Sample Characteristics

**Figure 1:**
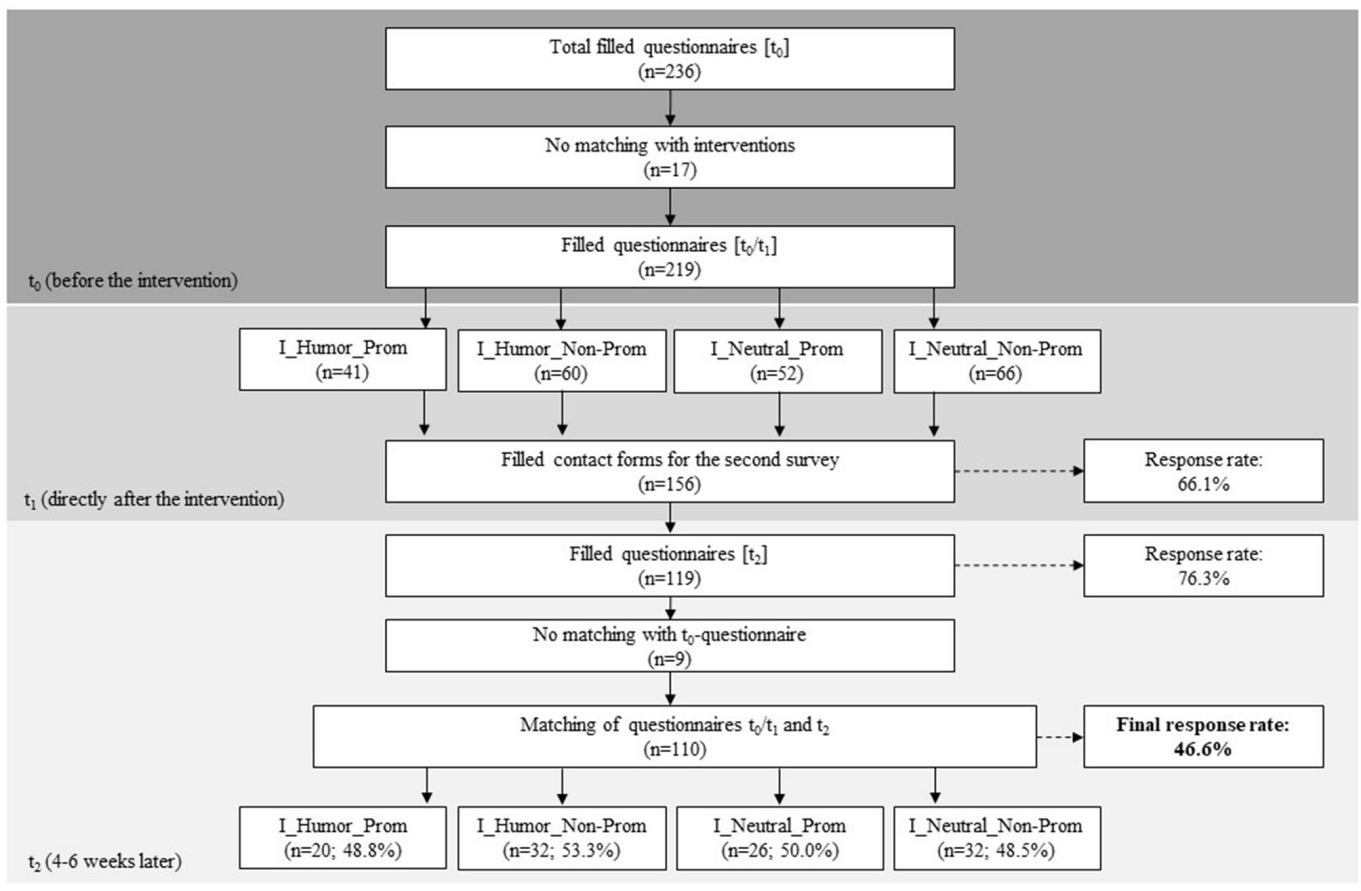
Flow chart for recruitment.

A total of 236 students participated in the first survey. However, only 219 watched the full video sequence and were, therefore, clearly assigned to one of the four interventions. This sample of 219 students was the one used for all further analyses. After this first survey, 156 students provided their email address. Four to six weeks later, 119 students completed the questionnaire for t_2_. This represents a response rate of 76.3%. Of these 119 questionnaires, 110 could be matched to an existing t_0_/t_1_ questionnaire. Therefore, the final response rate is 46.6% (Figure 1).

In the total sample (*n* = 219), 77.2% of the study participants were female. The mean age at baseline (t_0_) was 24.60 years (SD = 5.86). Most of the study participants were enrolled in social or educational sciences (37.9%), followed by mathematics, natural or engineering sciences (21.0%), and a much smaller proportion in medicine, public health, or nursing sciences (4.6%). Due to the overall small sample sizes in the subgroups, we did not conduct any statistical tests to assess the distribution. However, the distribution of age and gender across the intervention groups is comparable according to the descriptive results (Table 1).

**Table 1:** Sample characteristics at baseline (*n* = 219).

	**Intervention**	**I_Humor_Prom**		**I_Humor_Non-Prom**		**I_Neutral_Prom**		**I_Neutral_Non-Prom**		**Total**	
		***n***	**%**	***n***	**%**	***n***	**%**	***n***	**%**	***n***	**%**
	***Total***	**41**	**100**	**60**	**100**	**52**	**100**	**66**	**100**	**219**	**100**
Gender	Male	6	14.6	15	25.0	16	30.8	12	18.2	49	22.4
	Female	35	85.4	45	75.0	36	69.2	53	80.3	169	77.2
	Diverse	–	–	–	–	–	–	1	1.5	1	0.4
Age (in years)	<22	12	29.3	18	30.0	17	32.7	20	30.3	67	30.6
	22–25	21	51.2	29	48.3	20	38.5	25	37.9	95	43.3
	>25	8	19.5	13	21.7	15	28.8	21	31.8	57	26.0

### Non-Response Analysis

A non-response analysis was conducted to investigate whether the participants who only participated at t_0_/t_1_ differed from those who completed t_0_/t_1_ and t_2_. There were no statistically significant differences between these two groups. Only when looking at (non-)responders differentiated by intervention, was one significant result found: In I_Neutral_Prom, individuals who possessed an organ donor card took part significantly more often at t_2_ than their counterparts without donor cards [$\chi_{\left(\text{df}=1\right)}^{2}$ = 4.28, *p* = 0.039].

### Baseline Characteristics Related to Organ Donation

In the overall study population (*n* = 219), over three-quarters of students had a rather positive attitude toward organ donation (75.8%, *n* = 166), while 7.3% (*n* = 30) had a rather negative attitude at t_0_, and 15.9% (*n* = 35) described their own attitude as neutral or had not yet thought about the issue of organ donation. Overall, this general attitude was comparable between the participants in all four intervention groups. However, the general attitude toward organ donation was slightly, but not significantly, better in the sub-group of participants (*n* = 110) who took part at both t_0_/t_1_ and t_2_ (rather positive: 80.9%, *n* = 89; neutral: 13.6%, *n* = 15; rather negative: 5.5%, *n* = 6).

Within the total sample, 64.7% (*n* = 142) possessed an organ donor card at t_0_, of whom 92.3% (*n* = 131) agreed in principle to organ donation. Differentiated by gender, the percentage of women with organ donor cards is larger, at 69.8% (*n* = 118), than that of men, at 46.9% (*n* = 23), showing a statistically significant difference [$\chi_{\left(\text{df}=1\right)}^{2}$ = 8.70, *p* = 0.003]. Intuitively, when differentiated by general attitude, it was found that 91.5% (*n* = 130) of those with an organ donor card were likely to have a positive attitude toward organ donation compared with those without an organ donor card. In contrast, of those without an organ donor card, 46.8% (*n* = 36) had a positive, 41.6% (*n* = 32) a neutral, and 11.7% (*n* = 9) a negative attitude toward organ donation [$\chi_{\left(\text{df}=2\right)}^{2}$ = 59.0, *p* < 0.001].

Study participants were asked about their agreement with prodonation and antidonation statements at t_0_. Four out of the five prodonation statements showed a similar picture in terms of strong agreement: 94.0% (*n* = 206) of students agreed or strongly agreed with the statement that organ donation “helps other people in need of organs” (M = 5.72, SD = 0.81) and 89.1% (*n* = 195) agreed or strongly agreed with the statement: “I would be glad to receive an organ myself if needed” (M = 5.54, SD = 1.01). Additionally, 91.8% (*n* = 201) supported the opinion that an organ donation is meaningful, because “many people are currently waiting for an organ and their life depends on an organ transplantation” (M = 5.55, SD = 0.87) as well as 81.3% (*n* = 178) agreeing that “I do not need my organs after death” (M = 5.23, SD = 1.22). When it comes to the statement “Organ donation makes sense because it gives meaning to my death,” the picture is less uniform: 22.0% (*n* = 48) of respondents agreed or fully agreed with this statement, 19.6% (*n* = 43) agreed to a lesser extent, whereas 21.0% (*n* = 46) tended to disagree and 37.5% (*n* = 82) showed full disagreement with this statement (*M* = 3.12, SD = 1.65). Students expressing a higher level of overall agreement with the prodonation scale were also more likely to agree with organ donation (U = 153.500, *z* = −4.619, *p* < 0.01).

The most pronounced antidonation arguments are the fear of misuse (M = 2.96, SD = 1.48) and the fear of not being dead when organs are removed (M = 2.92, SD = 1.62). In descending order, this is followed by the fear that hospital staff will not work as hard to save patients' lives if they have an organ donor card (M = 2.59, SD = 1.52), the opinion that brain death is not the death of a human being (M = 2.40, SD = 1.35), the fear of disfigurement of the body due to organ donation (M = 2.37, SD = 1.34), not wanting to be confronted with topics related to death (M = 2.37, SD = 1.42), and feeling unsuitable for donating organs (M = 2.19, SD = 1.27). Finally, and with a larger gap, the least pronounced reason for antidonation was: “If there is a life after death, maybe I need all my organs for it” (M = 1.52, SD = 0.95). Study participants with a negative attitude toward organ donation were more likely to agree with all the antidonation items, except for the statement “I do not want to be confronted with topics related to death.” This causes a statistically significant difference between students with negative attitudes toward organ donation compared to students willing to donate organs and in the overall agreement, as seen in the antidonation scale (U = 258.00, *z* = −3.945, *p* < 0.01).

Small variations—in prodonation and antidonation sentiments—of participants completing t_0_/t_1_ and t_2_ stratified by the intervention groups can be read in Tables 2, 3, which describe the changes in agreement from t_0_ to t_2_ in terms of the means for each item.

**Table 2:** Prodonation items at t_0_ and t_2_ (*n* = 110).

**Organ donation is meaningful, because**		**t**_****0****_		**t**_****2****_		**Parametric tests**			
		**M**	**SD**	**M**	**SD**	**M_**Diff**_**	***t***	***p*-value^**a**^**	**Cohen's *d***
I_Humor_Prom	…it helps other people in need of organs.	5.55	1.19	5.85	0.36	0.30	−1.10	0.285	0.246
	…it gives meaning to my death.	3.20	1.67	3.30	1.17	0.10	−0.41	0.681	0.093
	…I do not need my organs after death.	4.90	1.33	5.15	1.22	0.25	−1.22	0.234	0.275
	…I would be glad to receive an organ myself when needed.	5.90	0.30	5.85	0.48	−0.05	1.00	0.330	0.223
	…many people are currently waiting for an organ and their life depends on an organ transplantation.	5.45	1.05	5.55	0.82	0.10	−0.69	0.494	0.156
	*Prodonation scale*	5.00	0.79	5.14	0.61	0.14	−1.37	0.185	0.308
I_Humor_Non-Prom	…it helps other people in need of organs.	5.84	0.44	5.88	0.33	0.04	−0.44	0.662	0.078
	…it gives meaning to my death.	3.28	1.52	3.69	1.67	0.41	−2.14	0.040^*^	0.378
	…I do not need my organs after death.	5.00	1.43	5.13	0.87	0.13	−0.64	0.525	0.114
	…I would be glad to receive an organ myself when needed.	5.56	0.71	5.66	0.60	0.10	−1.13	0.263	0.202
	…many people are currently waiting for an organ and their life depends on an organ transplantation.	5.50	0.71	5.72	0.52	0.22	−1.48	0.147	0.263
	*Prodonation scale*	5.04	0.54	5.21	0.58	0.17	−2.98	0.006^*^	0.527
I_Neutral_Prom	…it helps other people in need of organs.	5.92	0.70	5.96	0.19	0.04	−1.00	0.327	0.194
	…it gives meaning to my death.	3.73	1.75	3.92	1.52	0.19	−0.69	0.495	0.136
	…I do not need my organs after death.	5.58	0.78	5.56	0.62	−0.02	−1.14	0.161	0.283
	…I would be glad to receive an organ myself when needed.	5.81	0.63	5.81	0.80	0	0	1.000	0
	…many people are currently waiting for an organ and their life depends on an organ transplantation.	5.65	0.48	5.73	0.53	0.08	−0.81	0.425	0.159
	*Prodonation scale*	5.34	0.58	5.42	0.60	0.08	−1.14	0.266	0.223
I_Neutral_Non-Prom	…it helps other people in need of organs.	5.69	1.12	5.69	0.96	0	0	1.000	0
	…it gives meaning to my death.	3.19	0.96	3.69	1.63	0.60	−2.49	0.018^*^	0.440
	…I do not need my organs after death.	5.38	1.26	5.34	1.23	−0.04	0.23	0.813	0.042
	…I would be glad to receive an organ myself when needed.	5.41	1.36	5.31	1.35	−0.10	1.35	0.184	0.241
	…many people are currently waiting for an organ and their life depends on an organ transplantation.	5.59	0.97	5.53	1.01	−0.06	0.57	0.572	0.102
	*Prodonation scale*	5.05	1.01	5.11	1.07	0.06	*-*0.73	0.470	0.129

a *p-value is based on t-test for paired samples.*

* *p < 0.05*.

**Table 3:** Antidonation items at t_0_ and t_2_ (*n* = 110).

**Fears and caveats**		**t**_****0****_		**t**_****2****_		**Parametric tests**			
		**M**	**SD**	**M**	**SD**	**M_**Diff**_**	***t***	***p*-value^***a***^**	**Cohen's *d***
I_Humor_Prom	I feel unsuitable for donating organs.	2.40	1.42	2.25	1.33	−0.15	0.64	0.527	0.144
	Organ donation disfigures the body.	2.00	1.25	2.45	1.27	0.45	−2.13	0.046^*^	0.476
	I am afraid that the hospital staff will not work as hard to save my life when I have an organ donor card.	2.35	1.42	2.25	1.55	−0.10	0.69	0.494	0.156
	I am afraid of misuse related to donated organs.	2.90	1.58	2.40	1.42	−0.50	2.23	0.038^*^	0.500
	I am worried that I am not really dead when organs are removed.	3.10	1.80	3.05	1.87	−0.05	0.27	0.789	0.061
	In my opinion, brain death is not the death of a human.	2.45	1.43	2.55	1.57	0.10	−0.43	0.666	0.098
	I do not want to be confronted with topics related to death.	2.45	1.23	2.60	1.35	0.15	−0.90	0.379	0.201
	If there is a life after death, maybe I need all my organs for it.	1.95	1.19	1.75	1.02	−0.20	1.45	0.163	0.325
	*Antidonation scale*	2.45	0.98	2.41	1.01	−0.03	0.20	0.636	0.107
I_Humor_Non-Prom	I feel unsuitable for donating organs.	2.25	1.27	1.97	0.86	−0.28	1.27	0.213	0.225
	Organ donation disfigures the body.	2.38	1.28	2.06	1.04	−1.68	1.71	0.096	0.304
	I am afraid that the hospital staff will not work as hard to save my life when I have an organ donor card.	2.69	1.44	2.69	1.30	0	0	1.000	0
	I am afraid of misuse related to donated organs.	3.09	1.46	3.06	1.45	−0.03	0.19	0.851	0.033
	I am worried that I am not really dead when organs are removed.	3.03	1.55	3.00	1.39	−0.03	0.22	0.823	0.040
	In my opinion, brain death is not the death of a human.	2.41	1.10	2.53	1.31	0.12	−1.00	0.325	0.177
	I do not want to be confronted with topics related to death.	2.53	1.36	2.63	1.36	0.10	−0.59	0.557	0.105
	If there is a life after death, maybe I need all my organs for it.	1.53	0.98	1.66	1.00	0.13	−1.00	0.325	0.177
	*Antidonation scale*	2.49	0.78	2.45	0.81	−0.04	0.61	0.547	0.108
I_Neutral_Prom	I feel unsuitable for donating organs.	1.77	0.95	2.04	0.95	0.27	−1.78	0.090	0.346
	Organ donation disfigures the body.	2.00	1.02	1.88	0.90	−0.12	0.59	0.559	0.116
	I am afraid that the hospital staff will not work as hard to save my life when I have an organ donor card.	2.12	1.21	2.04	0.99	−0.08	0.40	0.691	0.079
	I am afraid of misuse related to donated organs.	2.42	1.02	2.35	0.97	−0.07	0.37	0.713	0.073
	I am worried that I am not really dead when organs are removed.	2.35	1.19	2.23	1.03	−0.12	0.64	0.523	0.127
	In my opinion, brain death is not the death of a human.	1.88	0.99	2.19	1.20	0.21	−1.87	0.073	0.368
	I do not want to be confronted with topics related to death.	2.46	1.52	2.58	1.52	0.12	−0.53	0.600	0.104
	If there is a life after death, maybe I need all my organs for it.	1.27	0.66	1.50	0.90	0.23	−2.28	0.031^*^	0.449
	*Antidonation scale*	2.03	0.63	2.10	0.69	0.07	−0.81	0.423	0.159
I_Neutral_Non-Prom	I feel unsuitable for donating organs.	2.66	1.59	2.22	1.45	−0.44	2.30	0.028^*^	0.407
	Organ donation disfigures the body.	2.25	1.29	1.84	1.01	−0.41	1.89	0.068	0.334
	I am afraid that the hospital staff will not work as hard to save my life when I have an organ donor card.	2.31	1.42	2.09	1.05	−0.22	1.09	0.281	0.194
	I am afraid of misuse related to donated organs.	2.59	1.24	2.66	1.15	0.07	−0.38	0.701	0.069
	I am worried that I am not really dead when organs are removed.	2.81	1.49	2.66	1.45	−0.15	0.68	0.501	0.120
	In my opinion, brain death is not the death of a human.	2.41	1.36	2.41	1.31	0	0	1.000	0
	I do not want to be confronted with topics related to death.	2.09	1.20	2.28	1.25	0.19	−1.06	0.296	0.188
	If there is a life after death, maybe I need all my organs for it.	1.59	1.13	1.53	0.76	0.06	0.27	0.782	0.050
	*Antidonation scale*	2.34	0.84	2.21	0.62	−0.13	1.19	0.241	0.211

a *p-value is based on t-test for paired samples.*

* *p < 0.05*.

### Short-Term Effects of the Interventions (t_0_ to t_1_)

The humorous interventions (I_Humor_Prom: M = 7.15, SD = 2.03; I_Humor_Non-Prom: M = 5.78, SD = 1.89) were perceived as more entertaining than the neutral messages by those participants taking part in t_0_/t_1_ and t_2_ (I_Neutral_Prom: M = 4.15, SD = 1.84; I_Neutral_Non-Prom: M = 4.13, SD = 1.73) [F_(3, 106)_ = 14.57, *p* < 0.001].

Considering all the study participants (*n* = 219) who responded at t_0_ and t_1_ (irrespective of their later participation at t_2_), and summing up the results of all four interventions, because differences between the interventions in this regard are negligible, one can see that 33.4% (*n* = 73) stated that they had received new information due to the intervention, 42.9% (*n* = 94) claimed that they wanted to think about organ donation in the near future, and 58.9% (*n* = 129) wanted to talk with relatives and/or friends about organ donation. These changes are generally comparable to those in persons taking part in t_0_/t_1_ and t_2_ (*n* = 110), where agreement with the items listed above was 28.2% (*n* = 31), 44.5% (*n* = 49), and 63.6% (*n* = 70), respectively. Furthermore, there are no significant differences between the intervention groups in these responses.

In the total sample (*n* = 219), both viewed as a whole and differentiated according to intervention groups, the proportion of people with a positive attitude toward organ donation decreased slightly, whereas the proportion of people with a neutral attitude increased. Significant changes only occurred in the two intervention groups with a neutral message: In I_Neutral_Prom, the proportion of participants with a positive attitude decreased (86.5–76.9%), and in contrast the proportion of participants with a neutral attitude increased (11.5–21.2%) (Wilcoxon signed-rank test: *z* = −2.236; *p* = 0.025). The intervention group I_Neutral_Non-Prom showed a similar picture: The proportion of people with a positive attitude decreased from 74.2 to 59.1%. At the same time, the proportion of individuals with neutral attitudes increased from 15.2 to 31.8%. The proportion of individuals with negative attitudes decreased from 10.6 to 9.1%. The changes in this intervention group are also statistically significant (Wilcoxon signed-rank test: *z* = −2.183; *p* = 0.029).

### Long-Term Effects of the Interventions (t_0_ to t_2_)

The following results are based on those study participants from whom data were available at both t_0_ and t_2_ (*n* = 110). Therefore, the values on general attitude differ from those presented above related to the short-term effects.

More than 80% were able to remember the video presented by the non-prominent person (I_Humor_Non-Prom and I_Neutral_Non-Prom), whereas a lower proportion of 70.9% (I_Humor_Prom) and 73.1% (I_Neutral_Prom) remembered the video sequence presented by Dr. Eckart von Hirschhausen.

In contrast to the declining acceptance of organ donation after viewing the interventions as a short-term effect, the proportions having a generally positive attitude (80.9–81.8%) and a neutral attitude (13.6–14.5%) increased in the sub-sample of people taking part at both t_0_/t_1_ and t_2_. According to this, the proportion of people with a negative attitude toward organ donation decreased from 5.5 to 3.6%. Neutral attitude did not increase in any of the intervention groups. Whereas, there were no changes for I_Neutral_Prom (92.3% positive and 7.7% neutral), for I_Humor_Prom there was a change of 10 percentage points from a positive (85.0–75.0%) to a neutral (10.0–20.0%) attitude (Wilcoxon signed-rank test: *z* = −1.00, *p* = 0.625). In contrast, the general attitude was better at t_2_ than at t_0_ in the interventions with the non-prominent speaker, both for the humorous message (I_Humor_Non-Prom: 65.9–71.9% positive; stable 25.0% neutral; 9.4–3.1% negative) (Wilcoxon signed-rank test: *z* = −1.414, *p* = 0.157) and the neutral one (I_Neutral_Non-Prom: 84.4–87.5% positive; 9.4–6.3% neutral; stable 6.3% negative) (Wilcoxon signed-rank test: *z* = −0.378, *p* = 0.705). However, none of these changes was statistically significant. An increase in willingness to donate organs was found in both the humorous (McNemar test: *p* = 1.000) and neutral (McNemar test: *p* = 0.625) message characteristic groups, but neither change is significant.

In terms of reasons for or against organ donation, Table 2 describes changes in prodonation items and Table 3 changes in antidonation items from t_0_ to t_2_, stratified by intervention group. The only significant changes—with small effect sizes—were an increase in agreement with the statement that organ donation gives meaning to one's death within both interventions provided by the non-prominent speaker (I_Humor_Non-Prom: M_Diff_ = 0.41, *p* = 0.040, *d* = 0.378; I_Neutral_Non-Prom: M_Diff_ = 0.60, *p* = 0.018, *d* = 0.440). Three out of five items in I_Neutral_Non-Prom decreased slightly, whereas one stayed the same (“It helps others in need of organs”) and the last one—related to the meaning of death—increased significantly, as previously mentioned. For I_Neutral_Prom, almost no changes in the mean differences were observed. However, in both humorous interventions, all the mean values of the prodonation items increased, with the exception of the statement “I would be glad to receive an organ myself if needed” in I_Humor_Prom (Table 2).

For the antidonation items as well, only a few significant changes were observed. After the humorous intervention with the prominent speaker, two items on the antidonation scale changed statistically significantly. While there was a non-intended increase in agreement with the statement that an organ donation disfigures the body (M_Diff_ = 0.45, *p* = 0.046, *d* = 0.476), the fear of misuse related to donated organs decreased (M_Diff_ = −0.50, *p* = 0.038, *d* = 0.500). Responses in the humorous intervention provided by the non-prominent person also showed mixed effects without any significant changes (Table 3).

The scientifically neutrally formulated interventions each showed intended changes in four out of the eight items on the antidonation scale. However, a non-intended significant increase was observed in agreement with the statement “If there is a life after death, maybe I need all my organs for it” (M_Diff_ = 0.23, *p* = 0.031, *d* = 0.449) (Table 3).

## Discussion

This study reveals mixed results regarding the intervention's effects on attitudes relating to organ donation. Appraisals beneficial to organ donation improved significantly only in the non-humorous interventions in terms of giving meaning to one's death. Although the humorous interventions were perceived as more entertaining, their effects on attitudes related to organ donation were limited. In relation to antidonation, the humorous intervention presented by the prominent person only showed an intended significant effect in reducing the fear of misuse related to donated organs. However, the effects are inconsistent, because there is no clear direction of effect for all items. Furthermore, it seems to be of little relevance whether the humorous message was communicated by a prominent or non-prominent person. Overall, the four interventions led to high proportions of self-reported willingness to think about organ donation and talk about it with relatives and friends subsequent to the respective intervention.

The proportion of study participants who documented a decision regarding organ donation on an organ donor card, at around 65%, is almost twice as high as the corresponding proportion in a representative national survey in Germany (30). This can be explained due to the student sample, because the possession of an organ donor card and a positive attitude are associated with high levels of formal education and an age below 55 years (30–32). Overall, there was a strikingly high positive attitude (92.3%) toward organ donation among students possessing an organ donor card, whereas students who did not possess a card mainly had a neutral (41.6%) or even negative (11.7%) attitude. One possible explanation is that those without an organ donor card show a lower degree of involvement in the topic of organ donation. This may mean that they are unable or unwilling to form an opinion and, therefore, less likely to fill out an organ donor card (33). However, our study shows that fears and caveats related to organ donation are comparatively low in the group of University students.

In principle, the students seemed to be well-informed about organ donation, as a good two thirds of them stated that they had not received any new information from the video. After stratification according to the type of message (humorous vs. neutral), only marginal differences emerged. This confirms that obviously the same information was contained in both the neutral and humorous messages. At this point, the type of intervention appears to be of little relevance regarding information delivery and its effects on knowledge. However, the humorous messages were found to be statistically significantly more entertaining, particularly the one presented by Dr. Eckart von Hirschhausen. This can be attributed to his popularity as well as his experience in entertainment education. Nevertheless, the study highlights that the effects on willingness to donate organs and its determining factors in terms of prodonation and antidonation items do not uniformly or significantly differ when presented by a non-prominent person.

The differences in overall changes in general attitudes over the short term and long term may require further discussion. Immediately after the interventions, the proportion of people with a positive attitude decreased in all intervention groups. Various reasons for this are conceivable. On the one hand, one might argue that the arguments were not convincing. On the other hand, a cognitive process could have been stimulated and the information conveyed by the interventions had to be processed first (34, 35). It might be that fears and concerns were addressed (36) that study participants may not have been aware of beforehand. However, after processing the information, positive attitudes evolved among the study participants.

When distinguishing between humorous and neutral messages, it is noticeable that the changes are less pronounced among those who received the humorous messages compared to the neutral messages. The changes are statistically significant for both neutral interventions, indicating more critical information processing. In comparison, less critical information processing seems to have taken place following the humorous interventions (37). One might expect that the motivation to identify counterarguments was reduced, leading to an alleviated defensive reaction (38). However, it seems that the humorous messages were not able to unfold their full persuasive power (23).

Previous studies have investigated the effects of humorous interventions among the audience of a live medical cabaret on various health-related attitudes and behaviors (39, 40), including the same humorous intervention on willingness to donate organs (14, 15) which has been part of this study. Overall, one can conclude that humorous interventions were not *per se* more effective than neutral information (15), because the results were heterogeneous, showing small and even non-intended effects (14, 39, 40), as was also visible in our study results.

A recommendation based on previous studies (14, 15, 39, 40) was to examine the effectiveness of further media and information channels for presenting humorous interventions. For that reason, the present study investigated the extent to which a video sequence differed in its effects from the same intervention provided in a live medical cabaret. Overall, the effects within our study were somewhat less pronounced, because less significant changes in both the prodonation and antidonation items were observed, although effect sizes were even larger. The difference in effects could be attributable to various factors. For the effectiveness of medical cabaret, interaction with a live audience is a prerequisite (41, 42) in order to reach the target group with the message and enable emotional and cognitive processes due to a “positive captivity” (42–44). The humorous intervention based on a video sequence included an audience as well as their applause and laughter, but lacked the direct involvement of study participants. At the time of viewing the video sequences, the students were presumably without the company of others, and thus only passively involved. The shared positive laughter with many other people (43), which can lead to a more lasting anchoring of certain insights in memory (45), was therefore limited. This may have influenced the effectiveness of the humorous messages. A further reason for variations in effects is the fact that the humorous messages consisted of only an excerpt from the medical cabaret of <5 min. The advantage of a live show is the inclusion of many topics that can complement each other. Furthermore, the unique location of a live show, for example in a concert or event hall, offers a more intense and lasting experience (41).

In addition, one needs to consider that the student sample differed significantly from the audience of the live medical cabaret. For example, the average age of audiences at the live shows was around 45–50 years in previous studies (14, 15, 39, 40), and therefore differs significantly from the average age of about 25 years in this study. The variations in effects may be attributable to these heterogeneous samples. On the other hand, both samples (students and the audience of a live medical cabaret) mainly have a high level of education. This might explain the ceiling effect in that general attitudes related to organ donation were already very positive in the samples. However, the focus of interventions should be on the target group of people with lower levels of formal education, because they are less likely to possess an organ donor card or to have positive attitudes toward organ donation (30, 32).

Although this study was able to provide some evidence about the impact of humorous messages relating to organ donation, several questions still need to be addressed. Future studies should focus in detail on styles and traits of humor as well as individuals' involvement when evaluating the effects of humor in health communication. Furthermore, we have used a sample of University students which is representative of neither the whole population nor specific other sub-groups, who may profit from tailored communication strategies.

### Limitations

Overall, the response rate of 46.6% is good, but this only refers to the time between the first and second surveys. Due to the large number of universities that were contacted for this study, no overall response rate can be calculated. With regard to the content of the survey, it must be borne in mind that knowledge about organ donation and personal or professional experiences have not been included. Furthermore, we did not assess trait humor, therefore we could not investigate individual differences in the use and appreciation of humor. Despite using a standardized tool in which items related to prodonation and antidonation are theory-based, the information relied on self-reported data. This is of particular interest when interpreting the results related to information gain and the willingness to think or talk about organ donation later on. When interpreting the long-term effects of the interventions, one cannot rule out the possibility that changes in attitudes are affected by other factors than the intervention itself, because there has been a high media presence of the topic of organ donation at the same time.

All participants in the study were exposed to an intervention on the topic of organ donation. For future research, it would be of interest to form a control group that received either no intervention or an intervention containing information on a different topic. All study participants have been included in the analysis, independent of their prior attitude toward organ donation. This, in addition to the ceiling effect of an overall positive attitude already existing before the intervention, could have weakened the effects of the (humorous) interventions.

## Conclusions

This experimental study confirms the results of previous studies, according to which humorous communication—in the form of medical cabaret—on the one hand entertains the audience and on the other hand stimulates critical reflection on health-related topics. It shows that humorous interventions offer the potential to convey knowledge about organ donation and to address organ-donation-specific fears and caveats. Furthermore, this kind of intervention can also reinforce reasons for organ donation and support students in their positive attitude. However, it also contributes further important insights into measuring the effects of humorous interventions in health communication. The results indicate that the humorous intervention is not superior to a message with neutral characteristics. Furthermore, it reveals that the interventions can either be presented by a prominent or a non-prominent person, without impacting upon the intervention's effects. Future research should aim to increase our understanding of the symbols through which the rhetorical functions of humor manifest themselves in a variety of messages within health communication.

## Data Availability Statement

The raw data supporting the conclusions of this article will be made available by the authors, without undue reservation.

## Ethics Statement

The studies involving human participants were reviewed and approved by Ethics Committee of Bielefeld University. The patients/participants provided their written informed consent to participate in this study.

## Author Contributions

FF and RB conceptualized the study. RB collected and analyzed the data and drafted the manuscript. FF supervised this process and revised the manuscript critically for important intellectual content. All authors contributed to the article and approved the submitted version.

## Conflict of Interest

The authors declare that the research was conducted in the absence of any commercial or financial relationships that could be construed as a potential conflict of interest.
